# Assessment of dapagliflozin effect on diabetic endothelial dysfunction of brachial artery (ADDENDA-BHS2 trial): rationale, design, and baseline characteristics of a randomized controlled trial

**DOI:** 10.1186/s13098-019-0457-3

**Published:** 2019-07-31

**Authors:** Riobaldo M. R. Cintra, Alexandre A. S. Soares, Ikaro Breder, Daniel B. Munhoz, Joaquim Barreto, Sheila T. Kimura-Medorima, Pamela Cavalcante, Renata Zanchetta, Jessica Cunha Breder, Camila Moreira, Vitor W. Virginio, Isabella Bonilha, Jose Carlos Lima-Junior, Otavio R. Coelho-Filho, Vaneza L. W. Wolf, Gil Guerra-Junior, Daniela C. Oliveira, Rodrigo Haeitmann, Vicente H. R. Fernandes, Wilson Nadruz, Fernando R. P. Chaves, Carlos E. L. Arieta, Thiago Quinaglia, Andrei C. Sposito, ADDENDA-BHS2 trial investigators

**Affiliations:** 1 Cardiology Division, Faculty of Medical Sciences, State University of Campinas, Campinas, São Paulo 13084-971 Brazil; 2 Growth and Body Composition Lab, Center for Investigation in Pediatrics, Faculty of Medical Sciences, University of Campinas, Campinas, São Paulo Brazil; 3 Department of Ophthalmology, Faculty of Medical Sciences, University of Campinas, Campinas, São Paulo Brazil

**Keywords:** Diabetes, Endothelial function, SGLT2i, Dapagliflozin, Glibenclamide

## Abstract

**Background:**

Endothelial dysfunction (ED) is a hallmark in type 2 diabetes mellitus (T2DM) that favor both atherogenesis and ischemia and reperfusion injury (IRI). Sodium-glucose-2 co-transporter inhibitors (SGLT2i) may hypothetically improve microvascular and macrovascular functions via a broad spectrum of mechanisms, being superior to traditional antidiabetic therapy such as sulfonylurea, even in subjects under equivalent glycemic control. Hence, the present clinical trial was designed to compare the effect of these two treatments on markers of arterial wall function and inflammation in T2DM patients as well as on the potential mediating parameters.

**Method and results:**

ADDENDA-BHS2 is a prospective, single-center, active‐controlled, open, randomized trial. Ninety-eight participants (40–70 years old) with HbA1c 7–9% were randomized (1:1, stratified by gender, BMI and HbA1c levels) to either dapagliflozin 10 mg/day or glibenclamide 5 mg/day on top of metformin. The primary endpoint was the change of flow-mediated dilation (FMD) after a 12-week period of treatment evaluated at rest and after IRI between dapagliflozin and glibenclamide arms. Secondary outcomes were defined as the difference between treatments regarding: plasma nitric oxide (NO) change after FMD, plasma isoprostane, plasma levels of vascular inflammatory markers and systemic inflammatory markers, plasma levels of adipokines, anthropometric measures, glucose control parameters, office and ambulatory BP control. Safety endpoints were defined as systolic and diastolic function assessed by echocardiography and retinopathy change. Serious adverse events were recorded. The study protocol was approved by the Independent Scientific Advisory Committee.

**Conclusion:**

The ADDENDA-BHS2 trial is an investigator-initiated clinical trial comparing the effect of dapagliflozin versus glibenclamide on several aspects of vascular function in high cardiovascular risk T2DM patients. Besides, a large clinical and biochemical phenotype assessment will be obtained for exploring potential mediations and associations.

*Trial registration* Clinical trial registration: NCT 02919345 (September, 2016)

## Background

In type 2 diabetes mellitus (T2DM), endothelial dysfunction results from the direct effects of glucotoxicity, lipotoxicity, oxidative stress, insulin resistance [1] and inflammation [2]. This outcome is potentiated by increased sarcopenic obesity [3] and uncontrolled blood pressure (BP), which are commonly linked to diabetes fueling deterioration of endothelial function [4, 5].

In addition to modulating resting arterial tone, the endothelium plays a central role in controlling arterial flow after temporary occlusion, as in acute coronary syndrome. Decreased post-ischemic blood flow may be particularly vital in T2DM patients in whom ischemia and reperfusion injuries (IRI) are more pronounced and, as a consequence, myocardial infarctions are more extensive [6]. Increased susceptibility to IRI in the context of T2DM is due to several mechanisms, including mitochondrial dysfunction, increased oxidative stress stimuli and impairment of antioxidant capacities at several intracellular and extracellular locations.

Inhibitors of sodium-glucose co-transporter-2 inhibitors (SGLT2) have been shown to be effective in reducing blood glucose, systolic BP and improving body composition [7–10]. By inference, we may hypothesize that the sum of these effects can improve endothelial function, either by direct or indirect actions. Moreover, improvement of endothelial function may hypothetically mediate the reduction of IRI propensity. The present paper describes the study design, rationale, and baseline characteristics of a prospective, randomized and controlled trial that evaluated whether the addition of dapagliflozin improves endothelial function under resting conditions and after IRI when compared to sulfonylurea in patients with T2DM.

## Methods

### Study design and population

The Assessment of Dapagliflozin effect on Diabetic Endothelial Dysfunction of brachial Artery-Brazilian Heart Study 2 (ADDENDA-BHS2) trial (NCT 02919345) is a single-center, randomized, open, active-controlled, phase-4 trial. All volunteers signed an informed consent form before entering. Patients were recruited through advertisements in newspapers, radio and television. Clinical and laboratory measurements were performed at the Clinical Research Center (CRC) and Atherosclerosis and Vascular Biology Laboratory (AtheroLab) at UNICAMP. The description of the study follows the recommendations of the CONSORT statement.

### Selection criteria

Inclusion criteria were: (i) high cardiovascular risk, defined by stable coronary artery disease (CAD) or subclinical carotid atherosclerotic disease; (ii) T2DM taking up to two oral hypoglycemic agents; (iii) HbA1c between 7 and 9% at the time of randomization; and (iv) age between 40 and 70 years old. CAD was defined as previous myocardial infarction (MI) at least 6 months before inclusion or angiography showing > 70% stenosis in at least one coronary artery. Subclinical carotid atherosclerotic disease was defined by standard guidelines and diagnosed by the presence of [1] carotid plaque; [2] carotid Intima-Media Thickness (cIMT) ≥ 1 mm; or [3] or cIMT values > p75th for age, gender and race according to the distribution in the Brazilian population [11].

Exclusion criteria were: (i) contraindications to metformin use (estimated glomerular filtration rate (eGFR) calculated by CDK-EPI < 45 ml/min, AST or ALT > 3× upper reference limit); (ii) systolic BP (SBP) ≥ 140 or diastolic BP (DBP) ≥ 90 mmHg after 16 weeks of anti-hypertensive medication adjustment during run-in period; (iii) acute coronary syndrome, stroke or coronary artery revascularization within 6 months prior to enrolment; (iv) plasma triglycerides > 500 mg/dl; (v) polyuria, polydipsia, weight loss, or others clinical signs of volume depletion; (vi) insulin use; (vii) pregnancy or women during reproductive age; and (viii) refusal to participate or sign the Informed Consent Form. The informed consent was obtained by the principal investigator.

### Clinical care protocol

**Fig. 1:**
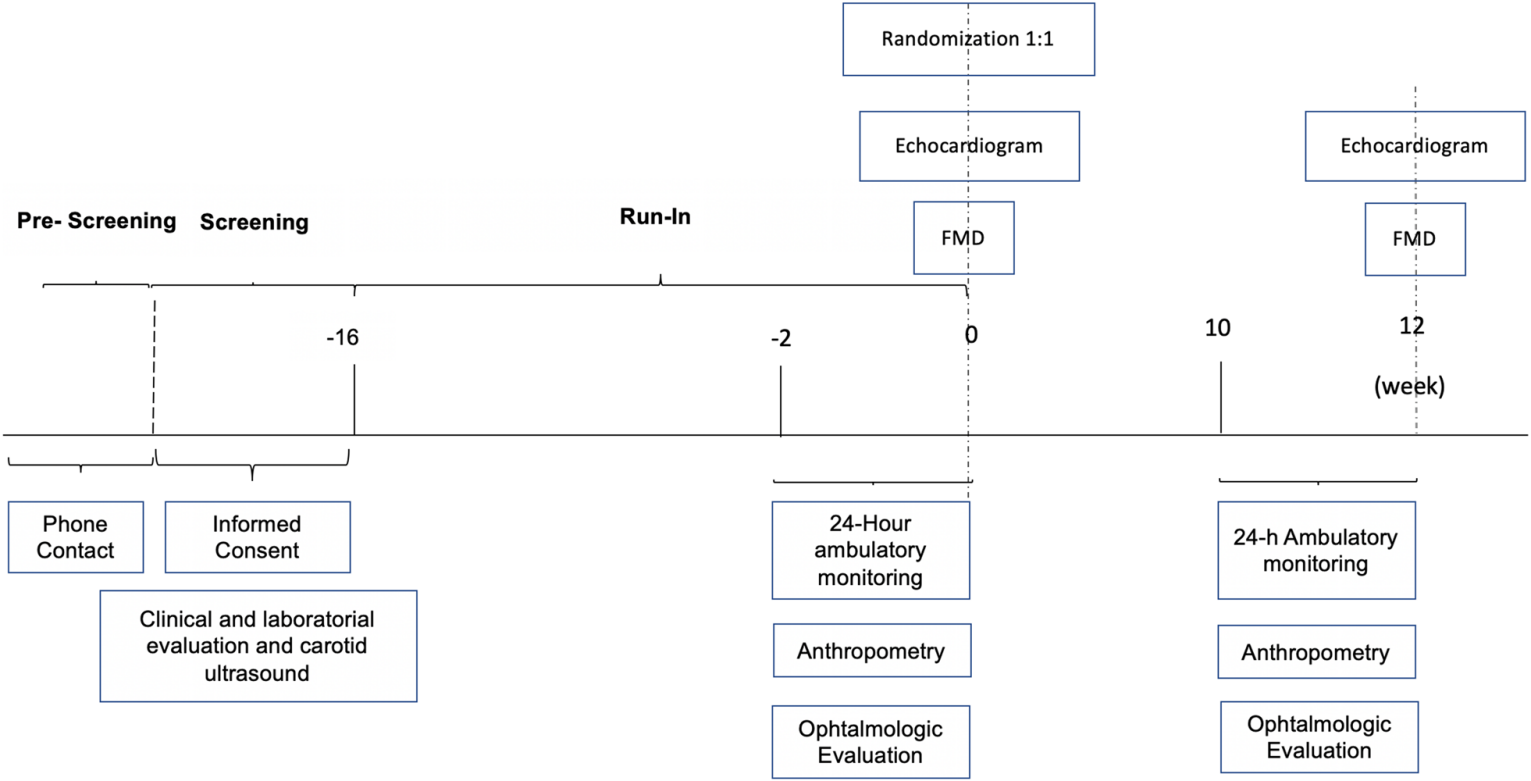
Study protocol

After assessing eligibility criteria by preliminary telephone interview, the volunteers underwent detailed clinical evaluation, anthropometry, carotid ultrasound, echocardiography, ophthalmologic evaluation and laboratory evaluation. Patients who fulfilled all the selection criteria above were submitted to a run-in phase to adjust the medications seeking at a HbA1c between 7 and 9% by the addition of extended-release metformin ≥ 1.5 g/day (Glifage XR Merck S.A., Brazil), and BP ≤ 130 × 80 mmHg, with losartan 25 to 100 mg/day (Aradois, Biolab-Sanus, Brazil) and the addition of hydrochlorothiazide 12.5 mg/day (Clorana, Sanofi, Brazil) when necessary. If after 16 weeks HbA1c values are < 7% or > 9% or BP is > 130 × 80 mmHg, patients will be discharged from the study. The Fig. 1 depicts the study protocol.

### Drug dispensing, adherence and adverse effects

At each visit, the study participants received a 30-day supply of their medications. On delivery of the medicines, patients were instructed to store the used blisters and bring them on the next visit. The number and lot of the pills were recorded on the Clinical Report Form (CRF), and the adherence was calculated based on the percentage of the medication that was taken. Drug adherence was stimulated by telephone calls conducted by a team member every fortnight. In this same telephone contact, patients were questioned about the manifestation of adverse effects and, if they had, advised to come to a medical visit for examination. These phone calls ensured the complete follow-up of enrolled patients. Characteristics and measures taken for adverse events were recorded in the CRF. Serious adverse events, whether or not considered causally related to the investigational therapy, were reported to the Institutional Ethics in Research Committee and AstraZeneca at the same time. As part of the protocol, patients with adverse events should contact the principal investigator and come to the hospital for evaluation shortly after reporting the event.

### Data management plan

Data were collected and managed during each visit at CRC using Research Electronic Data Capture (REDCap) tools hosted at Unicamp [12]. Clinical, blood pressure, electrocardiographic, ophthalmologic, anthropometric evaluations were obtained within a week before randomization and after experimental intervention. Echocardiography, laboratory and brachial artery evaluations were obtained on the day of randomization and the day of the 12-week visit. All research data collected is owned by the BHS group. The Principal Investigator of this project, Professor Sposito, takes responsibility for the collection and management of the research data. Study coordinator who is overseen by the Principal Investigator has provided quality assessment in a day-to-day basis. Over the course of the project, data have been collected and stored into the database center of the University following the best practices and standards and in accordance with REDCap rules. Digital video data files generated in the FMD experiments have been processed and stored in AVI format and the same database center. The principal investigators on the project and the BHS group will hold the intellectual property rights for the research data.

### Blood samples

After 12-h fasting period blood samples were obtained, centrifuged at 3500 rpm and thereupon measured for urea, creatinine, sodium, potassium, calcium, high-sensitive C-reactive protein (CRP), AST, ALT, glycemia, total cholesterol (TC), high-density lipoprotein cholesterol (HDL-C), and triglycerides (TG) (Cobas c702 Roche, Germany), insulin, thyroid stimulating hormone (TSH) (Cobas e602 Roche, Germany), blood count (Sysmex XN-L-Series, US). Low-density lipoprotein cholesterol (LDL-C) was calculated by a formula [13]. Urinalysis and urinary albumin/creatinine ratio (Dimension RXL MAX, Siemens, Brazil) were performed on the same day. Glycated hemoglobin (HbA1c) was measured by high-performance liquid chromatography (HPLC) (D-100, Bio-rad, Brazil). The Homeostasis Model Assessment version 2 (HOMA2) was used to estimate insulin sensitivity (HOMA2S) using fasting plasma insulin and glucose and calculator version 2.2.

At the randomization and the 12-week visit, blood samples were also obtained, centrifuged and frozen at liquid nitrogen in order to measure: plasma leptin, adiponectin (Invitrogen™ eBioscience™ ProcartaPlex Human leptin and adiponectin simplex kit, USA), Interleukin 6 (IL-6), Interleukin 2 (IL-2), tumour necrosis fact alpha (TNF-alpha), Vascular Cell Adhesion Molecule 1 (VCAM-1), Intercellular Adhesion Molecule 1 (ICAM-1) (Invitrogen™ eBioscience™ ProcartaPlex Human VCAM-1, ICAM-1, IL-6, IL-2, TNF-alpha Simplex kit, USA), Endothelin 1 (ET-1) (ET-1 Human ELISA Kit Thermo Scientific, USA), isoprostane (8-Isoprostane ELISA Kit, Cayman Chemical Company, USA).

### Anthropometry

Body composition was assessed by measures of weight, height, waist circumference, BMI and dual-energy X-ray absorptiometry (DXA) equipment (Lunar iDXA, GE Healthcare, USA). Briefly, a full-body scanner was performed with information on the body composition and bone mineral density of the femoral neck, Ward triangle, trochanter, and femoral body. The android fat mass was measured between the ribs and the pelvis, and the mass of gynoid fat was measured between the hips and the upper thighs.

### Blood pressure and electrocardiogram

Two clinic BP readings were taken at 60-s intervals after 3 min of rest using Omron HEM-705CP (Omron Healthcare, Japan) device, and their mean was defined as the office BP. The participant was then asked to stand up and an orthostatic reading was obtained after 3 min to assess for postural hypotension. Ambulatory blood pressure monitoring (ABPM) was measured as described elsewhere within a week before and after the 12-week experimental treatments (SpaceLabs, model 90207-8Q, USA). Electrocardiogram was recorded at baseline and after follow-up (Micromed Surface Digital Electrocardiogram, Brazil).

### Ophthalmologic evaluation

The best corrected visual acuity (BCVA) was tested, following the Early Treatment Diabetic test Retinopathy Study (ETDRS) in the distance evaluation, standardized to 4 m patient, and Jaeger’s table at close range, at a distance of 30 cm [14]. Biomicroscopy was performed on the anterior segment evaluation and, after maximum pupillary dilation (Mydryacil1% 2× e 1× Phelinefrine eye drops), patients underwent evaluation of the lens and retina. Lens opacification was classified based on Locs III [15]. Retina was appraised through posterior pole retinography by VISUCAM^®^ device (NM/FA Carl Zeiss, USA) and indirect ophthalmoscopy, following the International Classification of Diabetic Retinopathy and diabetic macular edema classification, proposed by Wilkison et al. [16].

Finally, patients underwent Optical Coherence Tomography-Spectral Domain (SD-OCT) macular examination, with the SPECTRALIS^®^ SD-OCT (Heidelberg Engineering, Inc., USA), according to International Council of Ophthalmology guideline classification [17]. The retinal layers were analyzed in the macular region, the thickness retinal region in the foveal region and the maximum thickness of the choroid in the subfoveal area was calculated, through the Enhanced Deep Imaging (EDI) method.

### Doppler ultrasound assessment of carotid arteries

Patients were screened for clinical or subclinical atherosclerosis by carotid arteries Doppler ultrasound with a 2.4–10 MHz linear array transducer (Vivid-S60, GE Healthcare, Milwaukee, Wisconsin). The patients were evaluated in a supine position with the head slightly extended and turned away from the side of the scanned carotid. After a transversal scan of the arteries, bilateral common, internal and external carotid arteries, as well as vertebral arteries, were imaged longitudinally, and ideally perpendicular to the transducer, by either anterior, lateral or posterior windows, whichever provided the highest image definition of the intima, media and adventitia layers. Images were recorded at frame rates of at least 20 per second and stored in a dedicated workstation for off-line assessments. Intima-media complex thickness (IMT) was assessed at end diastole 20 mm from the carotid bulb and at least 10 mm from the bifurcation. Focus was set to provide the best near and far wall resolution and at a depth of no more than 3–4 cm.

Atherosclerosis occurrence was defined as an IMT above the 75th percentile, IMT ≥ 1 mm, or the presence of plaque in the common carotid artery [11] as preconized by local guidelines. A plaque was defined as a localized protrusion of the arterial wall of more than 1.5 mm into the lumen or thickening of 50% of the arterial compared with an adjacent portion of the vessel wall. Apart from IMT evaluation, manual measurements of the intima, media and adventitia layers, separately, were performed after finding an optimized and zoomed still-frame image in order to individualize and estimate the thickness of each of these layers. These measures were made at the same spot defined for IMTs assessments. Doppler flow velocities of each artery and the respective resistivity index were calculated using the formula [(Peak systolic velocity-end diastolic velocity)/Peak systolic velocity].

### Analysis of endothelial function

Brachial arteries were measured using a high-resolution ultrasound (Vivid S6, GE Medical System, Milwaukee, WI, USA) according to previously published guidelines [18]. Briefly, the procedure takes place after over-night fasting and withdrawal of any vasoactive medications for the previous 24 h [19]. After 10 min of quiet resting in a supine position, and in a room with a controlled temperature around 22–25 °C, the brachial artery was located above the antecubital fossa, and a longitudinal image of 6 to 8 cm of the artery was considered as the baseline scan. The probe was then fastened to a probe holder (Quipu, Pizza, Italy) and sustained in an upright position. Continuous recording of real-time 2D image and simultaneous Doppler flow of the brachial artery was started by using a video capture device (Epiphan’s DVI2USB 3.0™, Epiphan Video, Ottawa, Canada) connecting the ultrasound to a dedicated computer. An appropriately sized blood pressure cuff was placed around the forearm and inflated up to 50 mmHg above the systolic blood pressure for 5 min and then deflated. The scan recording was obtained for 11 min (baseline: 1 min, ischemia: 5 min, and hyperemia and dilation: 5 min). Automatic edge-detecting software (LabVIEW 6.02, National Instruments) was used for online and off-line assessment of artery dilation and flow changes. The percentage change in diameter and flow for FMD was calculated in comparison with baseline scans. Other secondary parameters were also assessed offline by means of the same software, namely: the velocity–time integral of the antegrade and retrograde brachial flow, as determined by Doppler flow evaluation; the area under the curve (AUC) of the diameter versus time scatterplot; time to maximum vasodilation; and the derived parameters of blood flow (the product of cross-sectional area and Doppler velocity) and shear rate (4 times velocity divided by arterial diameter). These are validated methods used for covariate adjustment and microvascular endothelial function analysis. Two FMD assessments were performed in two different time-points—at the time of randomization and after 12 weeks of intervention (or control)—totalizing 4 FMDs for each patient. Fifteen minutes after the first FMD, reperfusion injury of the brachial artery was performed by cuff-induced ischemia (50 mmHg above the systolic blood pressure) for 15 min, followed by 15 min of reperfusion. At that moment, the second FMD analysis was repeated and the same parameters described above assessed.

Nitrite and nitrate (NOx) levels in plasma were measured just before FMD and 1 and 5 min after brachial artery cuff deflation. This collection was repeated during the second FMD after ischemia and reperfusion. Samples were collected in a tube containing heparin, centrifuged at 3500 rpm in a refrigerated centrifuge and stored in liquid nitrogen in up to 3 min for further analysis of NOx by a NO chemiluminescence analyzer (Model NOA, Sievers Instruments, Boulder, CO, USA).

### Echocardiography assessment

Patients underwent cardiac ultrasound analysis with a 1.5–4.5 MHz phased array transducer (Vivid-S60, GE Healthcare, Milwaukee, Wisconsin) on the day of the randomization and again at the end of the intervention (or control) periods. Still images and cine-loops were acquired, as necessary, for an off-line evaluation by an experienced physician blinded to patients’ characteristics and allocation to intervention or control arms. Cardiac chamber quantification was performed according to the current American Society of Echocardiography (ASE) Guidelines [20]. Left ventricular diastolic function status was determined by evaluation of tissue Doppler myocardial velocities (acquired in early diastole at the anterior, inferior, lateral and septal mitral annulus portions), mitral wave inflow velocities, body surface area-indexed left atrial volumes and tricuspid regurgitation peak velocities, as recommended by ASE guidelines [21]. Left atrial strain complemented left ventricular diastolic function evaluation as proposed recently [22]. Finally, the global longitudinal strain and post-systolic strain (PSS) were assessed by speckle tracking. All the exams were recorded and reviewed by three experienced echocardiographers and in the absence of consensus the evaluations were reprocessed.

### Randomization and follow-up

Randomization was made in 1:1 ratio on strata according to gender, baseline levels of HbA1c (7–7.9% or 8–9%) and BMI (below or above 30 kg/m^2^) for dapagliflozin (10 mg/day) or glibenclamide (5 mg/day) on top of metformin. The REDCap system was used for randomization at the study site by the principal investigator. Glibenclamide was selected as a comparator so that it could be based on the known effect and tested on the RCT with hard events in this population and thus estimate a potential gain with the new therapy [23–25]. The doses of dapagliflozin and glibenclamide were chosen in order to obtain similar magnitudes of reduction of glycemia. After randomization, patients were evaluated every 30 days in order to check clinical status, treatment adherence, adverse events, use of concomitant medications.

### Objectives and endpoints

The primary objective was the comparison of the FMD change at rest and after IR between a 12-week period of treatment with dapagliflozin or glibenclamide on top of metformin. Secondary outcomes were the difference between treatments regarding: (i) plasma NOx change after FMD; (ii) plasma isoprostane; (iii) plasma levels of the following vascular inflammatory markers ICAM-1, VCAM-1 (iv) plasma ET-1; (v) plasma levels of adipokines: adiponectin and leptin; (vi) systemic inflammatory markers: hs-CRP, TNF-alpha, IL-6 and IL-2; (vii) anthropometric measures: waist circumference, weight, body-mass index, body composition (% of fat mass and % free fat mass) and android/gynoid percent fat ratio, (viii) glucose control parameters: fasting glycemia, insulin, HOMA2S, HbA1c, (ix) office and ambulatory BP control, (x) systolic and diastolic function, and cardiac structure assessed by echocardiography, and (xi) retinopathy change during treatment.

### Sample size

Based on previous observations, we assumed a 3% change in FMD between arms (reduction of 1% in glibenclamide and increase of 2% in dapagliflozin), with a mean pre-treatment value of 5.5% and a standard deviation of 3.9% [26]. As we had a double primary outcome, i.e. rest and post-IRI FMD, we performed the Bonferroni’s method of adjustment and, hence, considered an alpha value of 0.025 and beta level of 90%. Under these conditions, it would take 44 patients per arm to achieve the necessary statistical power. However, as the researchers took into account that this study has a large number of secondary endpoints, the total sample was defined in 98 individuals, 49 in each arm. The sample size was calculated with G*Power 3.1 Mac version (Heinrich-Heine-Universität, Germany).

### Statistical analyses

Estimates of change from baseline will be compared between treatments for each primary endpoint. Results will be analyzed on an intention to treat principle. This study will be considered a success if one or both of the primary endpoints differ between treatment groups with statistical significance. Similar comparisons of secondary endpoints will be made with adjustment for multiplicity, and statistical significance will be inferred at a two-sided Type I error level of 0.025. Adjustment for potential confounders such as statin use will be employed for the results analyses. The data are shown as the mean ± standard deviation (SD) for normal distribution or median and interquartile range (IQR) for non-normal distribution data. Baseline continuous and categorical data were compared by Wilcoxon–Mann–Whitney test and a two-tailed t test or Fisher exact test. Analyses were performed using the SPSS 22, Mac version (IBM, USA).

## Results

### Participant baseline characteristics

**Fig. 2:**
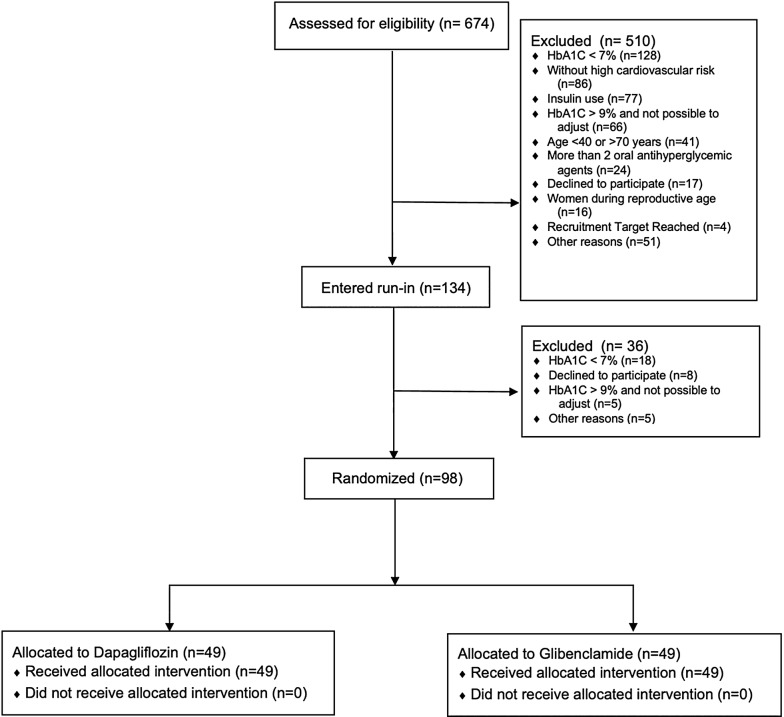
Flow diagram of the study

Recruitment of participants for the Addenda-BHS2 trial began in September 2016 and was completed in December 2018. A total of 674 subjects were evaluated and 134 met the selection criteria for the run-in phase when they underwent adjustment of medications and clinical control (Fig. 2). Of these, 98 individuals were enrolled into the trial. The main reasons for screen failure were HbA1c < 7% or > 9% (28.6%), low cardiovascular risk (12.8%), insulin use or age < 40 or > 70 years (6%). At the first visit, enrolled patients were using antidiabetic, antihypertensive and lipid-lowering medications in similar proportions (Table 1). At this time, lipid-lowering therapies were maintained at the same doses and were not introduced for those patients who were not yet in use to avoid interfering upon the primary endpoint of the study. Antidiabetic and antihypertensive medications were replaced with study drugs for a 16-week run-in period.

**Table 1:** Medications in use before the enrollment for the 16-week period of run-in

	Dapagliflozin	Glibenclamide	p
Metformin, %	96	98	1.0
Sulfonylureas, %	41	49	0.5
DPP-4i, %	4	14	0.16
Pioglitazone, %	2	0	1.0
ACEi, %	6	2	0.6
ARB, %	50	50	1.0
Statins, %	45	43	1.0

*DPP-4i* dipeptidyl peptidase-4 inhibitors, *ACEi* angiotensin-converting-enzyme inhibitor, *ARB* angiotensin-receptor blockers

The baseline characteristics of the participants are shown in Table 2. It is a population with a mean age of 57 years, most males (61%) and about 10 years of T2DM (median 8 and interquartile range of 10 years). Prior coronary artery disease was found in 8 patients (8%) and cIMT above the 75th percentile for Brazilian population was the criteria for 90 patients (92%). Consistent with a tight metabolic control, the majority (98%) had eGFR > 60 ml/min/1.73 m^2^ (57 to 142 ml/min/1.73 m^2^). All patients were normoalbuminuric (urinary albumin/creatinine ratio range from 0.01 to 2 mg/g). Systolic and diastolic blood pressure measured in the office were on average 137 and 82 mmHg, respectively, and 79% of the participants (77) were taking antihypertensive medication. The mean systolic and diastolic blood pressure obtained during 24-h ambulatory monitoring was 126 and 77 mmHg, respectively. The mean levels of LDL-C, HDL-C and triglycerides were 95 mg/dl, 41 mg/dl and 181 mg/dl, respectively. A total of 42 (43%) and 1 (1%) participants were taking statins and ezetimibe, respectively. Although no patient enrolled into the trial were active smoker, about 42% smoked in the past and stopped 19 ± 9 years ago.

**Table 2:** Characteristics of enrolled patients

	Dapagliflozin	Glibenclamide	p-value
N	49	49	
Gender male, %	61	61	1.0
Age, years	57 ± 7	56 ± 7	0.4
T2DM duration, years	9 ± 6	10 ± 7	0.28
Hypertension, %	82	76	0.6
Prior smoking habit, %	45	38	0.5
Sedentarity, %	55	65	0.4
Heart rate, bpm	71 ± 11	73 ± 10	0.28
Office systolic blood pressure, mmHg	136 ± 13	138 ± 15	0.67
Office diastolic blood pressure, mmHg	81 ± 8	83 ± 9	0.4
24-h systolic blood pressure, mmHg	124 ± 10	127 ± 11	0.1
24-h diastolic blood pressure, mmHg	72 ± 20	72 ± 21	0.88
Body mass index, kg/m^2^	30 ± 4	30 ± 5	0.76
Waist circumference, cm	104 ± 11	103 ± 10	0.32
Body fat, %	36 ± 8	37 ± 7	0.78
Android fat mass, g	2957 ± 1017	3203 ± 997	0.23
Gynoid fat mass, g	4168 ± 1518	4355 ± 1768	0.58
Retinopathy, %	15	28	0.2
Foveal diameter area by OCT, μm	267 ± 20	265 ± 23	0.69
Distal polyneuropathy, %	43	41	0.8
Fasting blood glucose, mg/dl	174 ± 44	173 ± 58	0.99
HbA1c, %	7.8 ± 0.9	7.9 ± 0.9	0.74
Fasting insulin, mU/l	14 ± 7	16 ± 14	0.53
Total cholesterol, mg/dl	163 ± 39	164 ± 42	0.99
LDL-cholesterol, mg/dl	93 ± 30	96 ± 35	0.76
HDL-cholesterol, mg/dl	42 ± 11	41 ± 10	0.62
VLDL-cholesterol, mg/dl	29 ± 15	28 ± 10	0.77
Triglycerides, mg/dl	179 ± 112	174 ± 94	0.76
TSH, mIU/l	2.2 ± 1.2	2.2 ± 1.7	0.67
Glomerular filtration rate, ml/min	96 ± 20	92 ± 19	0.39
Urinary albumin/creatinine ratio, mg/g	0.2 ± 0.2	0.3 ± 0.3	0.29
High-sensitivity C-reactive protein, mg/dl	0.1 ± 0.4	0.3 ± 0.6	0.08
Aspartate aminotransferase, U/l	23 ± 10	21 ± 12	0.46
Alanine aminotransferase, U/l	30 ± 17	29 ± 20	0.81
Carotid IMT, mm	1.0 ± 0.2	1.0 ± 0.1	0.15
tho, %	49	49	0.5
Carotid plaque, %	80	74	0.6
Left atrium volume indexed, ml/m^2^	24 ± 6	23 ± 4	0.2
Left ventricle end-diastolic volume, ml	84 ± 32	85 ± 28	0.96
Left ventricle mass indexed, g/m^2^	86 ± 22	88 ± 24	0.67
Left ventricle ejection fraction, %	65 ± 8	62 ± 7	0.29
Global longitudinal strain of the left ventricle, %	− 16 ± 8	− 15 ± 8	0.52
E/e′ ratio	7.9 ± 2.8	8.1 ± 2.2	0.99

## Discussion

The ADDENDA-BHS2 trial is a randomized, open-label, investigator-initiated clinical trial comparing the effect of dapagliflozin versus glibenclamide on endothelial function in high cardiovascular risk T2DM patients. We hypothesize that treatment with SGLT2i could have a favorable effect on both resting and post-IRI endothelial function as compared to a similar reduction of plasma glycemia by glibenclamide. In addition, the study was designed to explore as secondary endpoints the effect of the treatments on myocardial function, anthropometry, adipose tissue function, blood pressure and retina.

Endothelial function is influenced early and multifactorially by several cardiovascular risk factors, which generates an expectation of high predictive value for this test in risk estimation [27]. Indeed, endothelial dysfunction reveals clinically relevant conditions such as increased 10-year risk scores, microvascular angina and CAD severity [28, 29]. Accordingly, FMD has been used worldwide as a surrogate endpoint in prospective trials assessing the effect of different anti-diabetic and cardiovascular treatments [30–32]. However, the variability of FMD methods with induction of systematic errors and subjectivity in the discrimination of luminal edges have challenged its recognition as a reliable tool [33]. Automatic edge detection software allowing continuous recording of brachial artery diameter and guidelines for the method have greatly improved the reproducibility and repeatability of this technique. Still, endothelium evaluation by traditional FMD methods remains very preliminary, either mechanistically or as a surrogate endpoint, because it only gives access to a limited part of vascular physiology [19]. For instance, different populations have diverse time-to-peak dilation despite equal 1-min percent dilation [34].

This variability could be minimized by the ongoing method, in which four conditions were distinctly analyzed in a continuous manner: baseline, ischemia, hyperemia and vasodilation. This approach has several advantages over the traditional FMD protocol: (i) time-to-peak of dilation detection; (ii) peak dilation instead of 1-min single point dilation; (iii) area under the curve (AUC) for the whole extent of dilation; (iv) shear stress (doppler flow), (v) shear rate baseline (dyn/cm^2^), (vi) maximum shear rate (dyn/cm^2^), (vii) shear rate delta (0–100%), (viii) shear rate (AUC), (ix) antegrade flow (ml/min), (x) retrograde flow (ml/min), and (xi) oscillatory flow (ml/min). Analyzing several parameters in one experiment could reduce inter and intra-observer variation and, therefore, turn this method more accurate than traditional protocols.

During FMD, NO release is the main mediator of vasodilation concerning macrovascular function. The assessment of the role of NO in FMD has been made by using NO synthase blocker N(G)monomethyl-l-arginine (L-NMMA) [33]. In our approach, we performed a direct measurement of the bioavailability of NO in the brachial vein of the same arm submitted to FMD to estimate its role in altering arterial dilatation after treatment. Endothelial dysfunction is also associated with reduced FMD by increased ET-1 production [35]. The net balance between vasoconstrictor and vasodilatory stimuli will be assessed through the measurement of these molecules. It is also observed that the vascular endothelium phenotype could be addressed by its secretory profile [36]. Many vascular cytokines have a negative correlation to NO availability, such as the ones measured in this study: sICAM-1 and sVCAM-1.

This is the first clinical study assessing the effect of SGLT2i versus active comparator on IRI on equivalent glycemic control. Furthermore, although there is a broad spectrum of interaction between endothelial function and the magnitude of IRI, as mentioned above, this is the first study to simultaneously test endothelial function at rest and after IRI. In situations such as acute coronary syndromes, the behavior of the endothelium after IRI is certainly more relevant than that before acute vascular occlusion. Thus, we believe that our findings will have a greater potential for extrapolation to the potential clinical benefit of the intervention under investigation.

It has been shown that SGTL2i could alter the components of metabolic syndrome, such as BMI and waist circumference [37]. It is observed that both total and central adiposity relates to vascular dysfunction assessed by FMD [38]. In fact, SGLT2i could influence weight loss, and this change is pronounced compared to a sulphonylurea [39]. Moreover, it is possible that SGLT2i could promote visceral fat reduction [40], hence favoring vasodilation [41]. Due to the possible influence of weight and body composition on endothelium [42, 43], BMI, waist circumference and total adiposity evaluated by DXA will be measured. Also, adipose tissue function markers such as leptin, adiponectin and interleukin-6 have been reported to change after SGLT2i treatment [44] and to interplay on mediation of endothelial dysfunction [45]. Therefore, the measurement of these adipokines will be made on an attempt to estimate potential mediations.

In four recent clinical trials, hospitalization for heart failure (HF) was 30% less frequent in SGLT2i users than in the control groups [46–49]. This effect was equivalent in individuals with or without coronary artery disease or previous HF, suggesting the possibility of primary prevention of HF. In addition, in two small studies, treatment with SGLT2i improved diastolic function at short term [50, 51]. Among the mechanisms proposed are diuretic and hemodynamic action, improvement of myocardial metabolism and effects on cardiac and other ionic channels [52]. In individuals with type 2 or type 1 DM, endothelial function is directly associated with diastolic function [53–55]. It is not clear yet the mechanisms by which this association occurs but it is possible that the above-mentioned effects also influence both endothelial and diastolic functions. In fact, measures that improve endothelial function, such as the treatment of hypertension or dyslipidemia, also attenuate diastolic dysfunction [56, 57]. So far the interplay between left ventricle mechanics and the arterial tree buffering of stroke volume has not been addressed in this scenario. Assessing arterial and ventricular elastance, as well as, ventricular-arterial coupling would provide insight into mechanical efficiency of the transfer of blood from the heart to the arterial system. We hypothesize that dapagliflozin may improve ventricular-arterial coupling, LV systolic elastance and arterial elastance after 3 months of use.

The present study aims to test the effect of dapagliflozin versus glibenclamide on the attenuation of endothelial dysfunction in individuals with manifested or subclinical atherosclerosis. If a mitigation effect of endothelial dysfunction is found with the treatments tested in this study, our findings will help explain the reduction of cardiovascular events in patients with T2DM and established cardiovascular disease [46–49]. Our findings, however, should not be extrapolated to patients with less advanced T2DM duration in whom the development of endothelial dysfunction may not be fully established. Other studies should be done to estimate the effect of these drugs in preventing the decline of endothelial function over time.

## Conclusion

In summary, it is expected that the comparison of dapagliflozin with an established and widely used sulfonylurea adds to our current knowledge the description of mechanisms that may justify the reduction of the risk of macrovascular events with SGLT2i therapy. Also, the measurement of the clinical and biochemical profile may help in the understanding of mediators for the potential benefit of these treatments in endothelial function.

## Data Availability

The principal investigators on the project and the BHS group will hold the intellectual property rights for the research data. The datasets generated and/or analysed during the current study are not publicly available due to ongoing proprietary work but its availability would be considered by the corresponding author on reasonable request.

## Abbreviations

ED: endothelial dysfunction
T2DM: type 2 diabetes mellitus
IRI: ischemia and reperfusion injury
FMD: flow mediated dilation
NO: nitric oxide
SGLT2i: sodium/glucose cotransporter 2 inhibitor
ADDENDA: Assessment of Dapagliflozin effect on Diabetic Endothelial Dysfunction of brachial Artery
BHS: Brazilian Heart Study
CAD: coronary artery disease
MI: myocardial infarction
cIMT: carotid Intimia-Media Thickness
SBP: systolic blood pressure
DBP: diastolic blood pressure
CRF: clinical report form
CRP: C-reactive protein
TC: total cholesterol
HDL-c: high-density lipoprotein cholesterol
TG: triglycerides
TSH: thyroid stimulating hormone
LDL-c: low-density
HbA1c: glycated hemoglobin
HOMA2: homeostasis model assessment version 2
HOMA2S: homeostasis model assessment version 2 insulin sensitivity
IL-6: interleukin 6
IL-2: interleukin 2
TNF-alpha: tumour necrosis fact alpha
VCAM-1: vascular cell adhesion molecule 1
ICAM-1: intercellular adhesion molecule 1
ET-1: endothelin 1
BMI: body mass index
DXA: dual-energy X-ray absorptiometry (DXA)
BCVA: best corrected visual acuity
ETDRS: early treatment diabetic test retinopathy study
SD-OCT: spectral domain optical coherence tomography
EDI: enhanced deep imaging
IMT: Intima-media complex thickness
AUC: area under the curve
NOx: nitrate
ASE: American Society of Echocardiography
PSS: post-systolic strain
